# Nurse and parent perspectives of a neonatal intensive care unit redesign from open-bay to single-family rooms

**DOI:** 10.1038/s41372-025-02342-w

**Published:** 2025-07-03

**Authors:** Stephanie M. Teixeira-Poit, Bonnie Fields, Marjorie Jenkins, Susan Jones, Candace Matthews, Vannessa Gharbi, Serena Lowe, Frances A. Kendrick, sal ryman, Raven Funderburk

**Affiliations:** 1 Center of Excellence for Integrative Health Disparities and Equity Research (CIHDER), North Carolina Agricultural and Technical State University, Greensboro, NC USA; 2 John R. and Kathy R. Hairston College of Health and Human Sciences, North Carolina Agricultural and Technical State University, Greensboro, NC USA; 3 System-Wide Director of Nursing Research, Cone Health, Greensboro, NC USA; 4 Director of Neonatal Intensive Care Unit, Cone Health, Greensboro, NC USA; 5 Clinical Nurse Specialist, Children’s Services, Cone Health, Greensboro, NC USA; 6 School of Health & Human Sciences, University of North Carolina at Greensboro, Greensboro, NC USA; 7 Clinical Nurse, Neonatal Intensive Care Unit, WakeMed Health and Hospitals, Raleigh, NC USA; 8 Northern Michigan University, Marquette, MI USA; 9 College of Science and Technology, North Carolina Agricultural and Technical State University, Greensboro, NC USA

**Keywords:** Health services, Health policy

## Abstract

**Objective:**

To assess nurse and parent perspectives of a neonatal intensive care unit (NICU) redesign from open-bay (OPBY) to single-family rooms (SFR).

**Study design:**

We analyze interviews with NICU nurses and surveys with parents/guardians of neonates discharged from the NICU in the OPBY compared to SFR settings.

**Results:**

The SFR design increased privacy, eased facilitation of sterile and isolation procedures, and improved perceived comfort of parent participation in breastfeeding and kangaroo, or skin-to-skin, care. Increased privacy in the SFR design also resulted in unintended consequences including limited visibility of the healthcare team and increased need for clinician-parent communication. Policies and procedures meant to keep families safe during COVID-19 further decreased parents’ perceived access to and responsiveness of the healthcare team.

**Conclusions:**

Supportive policies and procedures promoting increased clinician-parent communication and additional parental supports may need to accompany transitions to SFRs to realize improvements in parental assessments of quality of care.

With over 400,000 preterm births in the United States (US) annually [1], neonatal intensive care units (NICUs) are critical to providing high-quality care and ensuring optimal health outcomes for preterm and sick neonates. NICUs began as open-bays (OPBYs) with multiple neonates admitted to large rooms of variable size. A growing trend in NICU design is single-family rooms (SFRs) with one neonate admitted per room and accommodations for at least one parent to stay with the neonate. This trend, partially fueled in the United States (US) by the 1996 Health Insurance Portability and Accountability Act’s emphasis on patient privacy and confidentiality, offers many clinical benefits, such as reduced infection rates and enhanced family involvement in the neonate’s care [2, 3]. Overall, the shift toward SFRs in NICUs supports family-centered care, an overarching goal to improve patient and family experiences in NICUs. In this study, we report both nurses’ and parents’ perceptions following a NICU design change from OPBY to SFRs, adding to the body of literature related to the important trend.

## Methods

### Study NICU context

This study assesses the efficacy of a NICU redesign from OPBY to SFRs within a flagship teaching hospital and referral center that is part of a larger health system in the Southeastern region of the United States. The NICU is a 45-bed department that provides care to premature and sick neonates 24 hours a day, seven days a week. The NICU in the study provides Level II and Level III care, including neonates 23 weeks and weighing less than one pound. Level II involves advanced care for neonates who are stable without complications but require special care and frequent feedings. Level III involves specialized care for neonates with high acuity conditions. The NICU has a high volume of patients with prematurity, respiratory distress, use of high frequency jet ventilation, hypoglycemia, and suspected sepsis.

The NICU initially had an OPBY design with multiple neonates admitted to multiple large rooms, with staff assigned based on patient acuity within the room. The OPBY NICU had an average staff of 65 full-time equivalent registered nurses (including on weekends) with 10.19 as the average number of registered nurse-hours per inpatient day. In 2020, a new Women’s and Children’s wing was constructed with a SFR design. In the first year, the SFR unit had an average staff of 71 full-time equivalent registered nurses (including on weekends) with 10.16 as the average number of registered nurse-hours per inpatient day. The first year also coincided with the COVID-19 pandemic, thus presenting a unique opportunity to examine the impact of the pandemic on unit design transitions. The results describe policy and procedure changes enacted in response to COVID-19.

### Data sources

Survey responses from parents/guardians of neonates discharged from the NICU in the OPBY versus SFR settings as well as interviews with NICU nurses were collected and analyzed for common themes.

### Survey data

The hospital contracted with a third-party vendor to administer satisfaction surveys to parents/guardians of neonates who were discharged from the NICU during a two-year period, including all data one year when the NICU had an OPBY design, and one year when the NICU had a SFR design. The data use agreement with the health system currently only allows the years of data presented in the manuscript. As noted elsewhere, in future works, plans are in place to obtain additional years of data.

The survey asked a series of questions about parent perspectives on care and clinician-parent communication using five-point Likert scales with the options (1) very poor; (2) poor; (3) fair; (4) good; and (5) very good. The survey evaluated parent perspectives including an overall assessment and feedback on nursing care, physician care, inclusiveness and responsiveness, and wait time. The survey also asked about parent feedback on communication including nurse-parent communication, physician-parent communication, and communication about tests and treatment.

#### Analytic technique

Survey data were imported into Stata Version 15.0 for analysis. Two-sided Wilcoxon-Mann-Whitney tests were used to compare ordinal level dependent variables from parents whose neonate received care in the OPBY design to those who received care in the SFR. The data meet the assumptions of the Wilcoxon-Mann-Whitney non-parametric test. Dependent variables are at the ordinal level, and observations in each group are independent of each other. Levene’s tests assessed homogeneity of variance and identified equal variances across groups for all outcomes. Both the medians and interquartile ranges are presented for each group for all outcomes. The code used to generate these results is available upon request.

We report statistically significant results as *p* < 0.05 and marginally significant results as 0.05 < *p* < 0.10. Using an a priori power analysis for a Wilcoxon-Mann-Whitney test conducted in G*Power, it was determined that a total sample size of 86 across two years, or 43 parents from each NICU design, is needed to achieve power for the analyses assuming alpha level of 0.05, power of 0.80, an effect size of 0.50, allocation ratio of 1.0, and two-tailed distribution. The parental surveys were an unmatched convenience sample with a total sample size of 88, including 55 in the OPBY design and 33 in the SFR design.

### Interview data

Qualitative semi-structured interviews were facilitated to determine perspectives of NICU nurses. A semi-structured interview guide was developed by: (1) drafting questions; (2) receiving input from academic team members and clinical partners; (3) revising the guide to address feedback; (4) pilot testing the interview guide; and (5) refining the guide based on lessons learned from the pilot testing. The interviews presented in the manuscript were conducted among nurses in the SFR setting, and almost all also had provided care in the OPBY setting. Interview questions asked nurses to reflect on their experience providing care in the OPBY setting, providing care in the SFR setting, and then comparing and contrasting the two settings. The interview protocol is provided in Supplemental File 1.

#### Recruitment

NICU nurses were recruited by: (1) emailing NICU staff about the interviews and linking to a screener to complete if interested in participating; (2) posting flyers with tear-off tabs containing a QR code to the screener; and (3) having NICU staff champions spread the word about the interviews. The screener was an online form that asked demographic questions. The NICU nurses who completed the screener were then emailed to schedule interviews. In an interview reminder email, respondents were asked to review a digital version of the informed consent. This research was approved by the university’s and healthcare system’s institutional review boards and the healthcare system’s nursing research council. All methods were performed in accordance with the relevant guidelines and regulations.

#### Data collection

A two-person team facilitated the interviews, including an experienced moderator and a notetaker. At the beginning of the interview, informed consent was obtained from all interviewees. Interviews followed a semi-structured interview protocol and lasted about 60 to 90 minutes. Respondents received $50 Visa gift cards for participating in the interviews. The interview sample included 11 percent of nurses who provided care on the NICU. All nurses were White, non-Hispanic women. Nurses varied in their experience in the NICU-care setting, ranging from one to more than 15 years of experience. A plurality of nurses had their bachelor’s degrees and worked between 30 to 39 hours per week. Nurses varied in the days and shifts worked in the NICU including those working weekdays, weekends, day shifts, and night shifts.

#### Coding process

Interviews were recorded with respondent permission and auto-transcribed via Zoom. Auto-transcriptions were manually cleaned to create verbatim transcripts, which were imported into NVivo for coding. Using coding best practices, a team of coders open coded the data to identify topics and used the results of the open coding to develop a codebook with code names, code definitions, examples of quotations, and inclusion/exclusion criteria. Interviews were conducted until code and meaning saturation were reached. Focused coding, interrater reliability assessment, consensus building, and codebook revision were performed in multiple rounds. In each round, two team members engaged in focused coding to code a subset of the transcripts using the codebook.

Interrater reliability was assessed between the two coders with the goal of achieving Cohen kappa coefficients of greater than 0.80 for each code, which signals high consistency of coding. In instances in which the coders achieved coding reliability of equal to or lower than 0.80 for a particular code, coders met to review the segments with differences in coding, discuss the rationale for their coding decisions, and reach consensus on coding decisions. This sometimes resulted in subsequent revisions to both the coding and codebook. After completing this process, coding reports were run on the coded data. Content analysis was conducted of coding reports to systematically analyze and identify patterns within and across codes and sub-populations.

## Results

### Nurse perspectives: single-family room (SFR) setting

#### Overall quality of care

Generally speaking, nurses reported that there was no difference in the quality of care provided in the OPBY compared to the SFRs. One nurse simply stated, “…everybody received the same quality of care.” Another indicated that, between the OPBY and SFR designs, “I don’t really feel like there was any difference in the care that patients or families received.” Yet another explained that, in the SFR design, “sterile procedure… has definitely improved.” She continued, indicating the unit went for quite some time without getting any central line associated blood stream infections (CLABSIs). A verbatim quote from another nurse is provided in Supplemental File 2 Table 1.

#### Benefits of SFRs

The level of privacy substantially increased when the NICU transitioned from OPBY to SFRs. In the SFR setting, nurses indicated that the increase in privacy provided more space, enhanced opportunities for parent visitation, and eased facilitation of sterile and isolation procedures. Verbatim quotes from nurses about the benefits of SFRs are provided in Supplemental File 2, Table 2.

Another benefit of increased privacy was the perceived comfort of parents to participate in breastfeeding and kangaroo, or skin-to-skin, care. Many nurses had opinions about the benefits of kangaroo care. Illustrative verbatim quotes are provided in Supplemental File 2, Table 2.

### Disadvantages of SFRs

While this increased privacy was perceived as positive, it also resulted in a lack of visibility of the nurse, which presented communication challenges. Verbatim quotes on disadvantages of SFRs and strategies to overcome them are provided in Supplemental File 2, Table 3.

While nurses generally reported that they provided the same level of care in both the OPBY and SFRs, nurses discussed changing their communication in the SFRs to proactively mitigate parents’ negative perceptions and ensure that families understood that the quality of care remained high. Nurses reported needing to “make a concerted effort to make that communication so that parents… know where you are on the unit.” Verbatim quotes from other nurses are provided in the Supplemental File 2, Table 3.

#### Impacts of the COVID-19 pandemic

The COVID-19 pandemic intensified shortly after the move to SFRs, and nurses reported its impact on several policies, processes, and procedures. In the OPBY setting, the medical team did rounds in person at the patient’s bedside, but this changed within the SFR setting. In the SFR, the medical team discussed patients and discussions were then relayed to nurses, families, and others, rather than the usual interdisciplinary rounds at the patient’s bedside. Not only did nurses express concerns related to parents and their access to the nursing staff in the SFR setting, but they also shared parental concerns about the access to other medical staff. These sentiments were expressed by many nurses (please see Supplemental File 2, Table 4 for verbatim quotes).

### Parent perspectives in the SFR compared to OPBY settings

Parent perspectives differed from nurses’ perspectives on overall quality of care. In parent surveys, parents’ overall rating of NICU care showed mixed results. In both the SFR and OPBY settings, the *median* overall rating of care given at the hospital, likelihood of recommending this hospital to others, rating of the skill of nursing care, and rating of the skill of physician care was *very good*. Yet, the percentage of parents who rated overall care as *very good* decreased from 73% in the OPBY setting to 58% in the SFR setting. Meanwhile, the percentage of parents who rated overall care as *very poor* decreased from 7% in the OPBY to 0% in the SFR.

Additionally, parents reported concern that their neonates’ needs were not always being met in the SFR. Parent perceptions of nurses paying *very good* attention to their special or personal needs lowered from 74% in OPBY to 52% in SFRs. Furthermore, the percentage of parents who rated nurse promptness in responding to alarms as *very good* lowered from 60% in the OPBY to 45% in SFRs. The median rating of nurse promptness in responding to alarms marginally decreased from 5.00 in OPBY to 4.00 in SFRs (*p* = 0.096; see Table 1).

**Table 1:** Parent Perspectives on Care.

Domains	Variable (N)	OPBY Design	SFR Design	OPBY Design	SFR Design	*p* value*
		% Very Good	% Very Good	Mdn Score out of 5.00 (IQR)	Mdn Score out of 5.00 (IQR)	
*Overall Assessment*	Overall rating of care given at hospital (*N* = 88)	72.73	57.58	5.00 (1.00)	5.00 (1.00)	0.208
	Likelihood of your recommending this hospital to others (*N* = 89)	67.86	60.61	5.00 (1.00)	5.00 (1.00)	0.467
*Nursing Care*	Skill of the nurses (*N* = 88)	65.45	60.61	5.00 (1.00)	5.00 (1.00)	0.611
	Nurses’ promptness in responding to alarms (*N* = 88)	60.00	45.45	5.00 (1.00)	4.00 (2.00)	0.096
	Amount of attention paid to your special or personal needs (*N* = 87)	74.07	51.52	5.00 (1.00)	5.00 (1.00)	0.052
*Physician Care*	Skill of physician (*N* = 87)	62.50	51.61	5.00 (1.00)	5.00 (1.00)	0.491
	Time physician spent with you (*N* = 86)	53.70	37.50	5.00 (1.00)	4.00 (2.00)	0.060
*Inclusiveness/Responsiveness*	Staff effort to include you in decisions about your treatment (*N* = 87)	53.70	45.45	5.00 (1.00)	4.00 (2.00)	0.270
	Response to concerns/complaints made during your stay (*N* = 86)	51.85	43.75	5.00 (1.00)	4.00 (1.50)	0.352
	Degree to which hospital staff addressed your emotional needs (*N* = 89)	57.14	51.52	5.00 (1.00)	5.00 (2.00)	0.526
	Degree to which hospital staff addressed your spiritual needs (*N* = 76)	57.14	44.44	5.00 (1.00)	4.00 (2.00)	0.253
*Wait Time*	Waiting time for tests or treatments (*N* = 87)	54.55	50.00	5.00 (1.00)	4.50 (1.00)	0.750
	Waiting time for results from your tests (*N* = 86)	54.55	38.71	5.00 (1.00)	4.00 (1.00)	0.282

* Wilcoxon-Mann-Whitney tests were used to compare ordinal level dependent variables from parents whose neonate received care in the OPBY design to those who received care in the SFR.

The percentage of parents who rated the time the physician spent with them as *very good* decreased from 54% in the OPBY to 38% in the single-family room. The median rating of time the physician spent with them marginally decreased from 5.00 in OPBY to 4.00 in the SFR (*p* = 0.060; see Table 1). Additionally, parent perceptions of nurses having *very good* attitudes toward requests lowered from 69% in OPBY to 49% in SFRs. Also, the percentage of parents who rated how well the physician kept them informed as *very good* lowered from 59% in the OPBY to 41% in SFRs. Both the median rating of nurse attitudes towards requests and how well physicians kept parents informed marginally decreased from 5.00 in OPBY to 4.00 in SFRs (*p* = 0.074 and *p* = 0.084, respectively; see Table 2).

**Table 2:** Parent Perspectives on Clinician-Parent Communication.

Domains	Variable *(N*)	OPBY Design	SFR Design	OPBY Design	SFR Design	*p* value*
		% Very Good	% Very Good	Mdn Score out of 5.00 (IQR)	Mdn Score out of 5.00 (IQR)	
*Nurse-Parent Communication*	Friendliness/courtesy of the nurses (*N* = 88)	72.73	54.55	5.00 (1.00)	5.00 (1.00)	0.122
	Nurses’ attitude toward your requests (*N* = 87)	68.52	48.48	5.00 (1.00)	4.00 (1.00)	0.074
	How well the nurses kept you informed (*N* = 88)	69.09	60.61	5.00 (1.00)	5.00 (1.00)	0.335
	Explanation of equipment on or near your child (*N* = 88)	69.09	48.48	5.00 (1.00)	4.00 (1.00)	0.101
*Physician-Parent Communication*	Physician’s concern for your questions and worries (*N* = 89)	57.14	45.45	5.00 (1.00)	4.00 (1.00)	0.290
	How well physician kept you informed (*N* = 88)	58.93	40.63	5.00 (1.00)	4.00 (2.00)	0.084
	Friendliness/courtesy of physician (*N* = 89)	64.29	57.58	5.00 (1.00)	5.00 (1.00)	0.634
*Tests and Treatments*	Explanations about what would happen during tests and treatments (*N* = 87)	60.00	56.25	5.00 (1.00)	5.00 (1.00)	0.945
	Courtesy of the person who took your blood (N = 82)	62.26	55.17	5.00 (1.00)	5.00 (1.00)	0.669
	Courtesy of the person who started the IV (*N* = 78)	57.69	65.38	5.00 (1.00)	5.00 (1.00)	0.609
	Communication about need for delays of procedure (*N* = 81)	55.77	48.28	5.00 (1.00)	4.00 (1.00)	0.677
	Courtesy of non-nursing staff when providing care or treatment for your infant (*N* = 87)	65.45	62.50	5.00 (1.00)	5.00 (1.00)	0.815
	Explanations regarding the purpose of tests or treatments (*N* = 87)	61.82	53.13	5.00 (1.00)	5.00 (1.00)	0.702

* Wilcoxon-Mann-Whitney tests were used to compare ordinal level dependent variables from parents whose neonate received care in the OPBY design to those who received care in the SFR.

### Nurse perspectives: open-bay (OPBY) setting

#### Disadvantages of OPBY

In the OPBY setting, several nurses reported that there was a lack of privacy for parents (please see verbatim quotes in Supplemental File 2, Table 5). To improve privacy during breastfeeding, the nurse sometimes proactively placed room dividers around the mother and neonate. The nurse also sometimes had to redirect parents to ensure that parents were not infringing on the privacy of other families. This reflected an effort to protect patient privacy and confidentiality—rights afforded by the US Health Insurance Portability and Accountability Act.

#### Benefits of OPBY

Although the lack of privacy had drawbacks, nurses noted that the resulting nurse visibility in the OPBY had some benefits for both nurses and parents. In the OPBY, the parent could visibly see the nurse providing care for not just their neonate, but also other neonates. One benefit of nurses being in the same room was increased parent confidence in nursing care because parents could see the nurse providing care to other neonates when not providing care to their neonate. Another benefit of nurses being in the same room was that families repeatedly heard education and discharge instructions given to other parents, allowing them to absorb information over time in the OPBY compared to the one time provided in SFRs. Many nurses articulated these sentiments, some of which are provided in the Supplemental File 2, Table 6. Yet, this lack of privacy was a concern for many nurses. While some nurses assumed increased privacy would increase parent visitation and parent-neonate interaction, they also acknowledged that parents who work might not have the same opportunities for visitation.

## Discussion

In this study, we report parent and nurse perspectives of care in a NICU that transitioned from OPBY to SFRs. The NICU redesign from OPBY to SFR resulted in several benefits and disadvantages. The increase in privacy in the SFR setting eased facilitation of sterile and isolation procedures and improved perceived comfort of parent participation in breastfeeding and kangaroo, or skin-to-skin, care. In the scholarly literature, both breastfeeding and kangaroo care are well documented as providing substantial benefits to preterm or low-weight neonates. In our sample, nurses clearly espoused breastfeeding and kangaroo care as part of their family-centered care approach.

These positive findings related to the increase in privacy also translated into less nurse visibility in the SFR design compared to the OPBY design. This finding is consistent with Doede et al.’s [4] work that found a tradeoff between privacy and visibility in the SFR setting [4]. In our study, the lack of nurse visibility resulted in nurses needing to change communication patterns with parents. The spatial layout of the SFR design also meant that nurses took longer to respond to alarms than in the OPBY. In the SFR design, nurses relied on Voceras [voice-driven smartbadges] to monitor neonates when they were not in the room. Taken together, it is likely that these disadvantages impacted parent perceptions of quality of care. While nurses generally reported that there was no difference in the quality of care, parent perceptions of overall care, nurse attention to special or personal needs, and nurse promptness to alarms decreased in the SFR compared to the OPBY. Our findings contrast Stevens et al. [3] who found that parents in SFRs had significantly higher ratings of the environment, overall assessment of care, and family-centered care than in OPBY [3].

Parent surveys revealed changes in clinician-parent communication with the SFR having lower ratings of nurse attitudes toward requests compared to the OPBY. One interpretation is that lack of nurse visibility in the SFR setting may be impacting parent perceptions of the quality of care. This interpretation is consistent with previous studies that found decreased visibility in the SFR, which increased nursing workload and had potential for adverse impacts on neonates [2]. In our study, nurse visibility in the OPBY design may have facilitated parent confidence in the nurse, however the lack of nurse visibility in the SFR setting may have required nurses to enhance communication to build parent confidence and trust. Thus, transitions to SFRs may result in substantial changes to nurse workflow and intra- and interprofessional collaboration and communication.

Furthermore, parents noticed a decrease in the time the physician spent with them in the SFR compared to the OPBY. Parent perspectives may reflect changes in policies, processes, and procedures implemented on the unit to ensure patient safety during the COVID-19 pandemic, such as changes to interdisciplinary rounds at the patient’s bedside and limitations to visitation. While these policies and procedures were restrictive in their nature and limited families’ ability to secure the usual support that exists among families in this setting, an important advantage was that SFRs offered protection from COVID-19 transmission. It is also important to note that, when studies examining transitions to SFRs found no difference in access to nursing staff or increased access to physicians, different policies (e.g., open visitation) were in place [5].

The SFR design helps hospital staff maintain privacy and confidentiality of patients as required under the 1996 Health Insurance Portability and Accountability Act. However, the OPBY design revealed some benefits to nurse visibility including parents’ ability to see the nurse providing care to other neonates when not providing care to their neonate, and families repeatedly hearing education and discharge instructions given to other parents. This finding was consistent with previous reports of parent satisfaction with the OPBY setting due to increased visibility of and communication with nursing and medical staff [5].

While SFRs offer benefits, our study revealed concerns about communication with and access to clinicians in SFR designs. Medical and nursing leadership can develop strategies to attenuate these issues. Potential strategies include: orienting families to job roles of the healthcare team; encouraging the healthcare team to communicate their unit location to families; using technology to support communications; developing brochures or reference sheets for families to outline various ways to reach the nurse (e.g., call light, telephone, unit secretary); developing orientation videos for families to share “A Day in the Life of the NICU Healthcare Team”; and providing additional opportunities for parent education about neonatal care and discharge.

One study limitation was that parental data used for statistical analysis was dependent on a parent’s choice to respond to an anonymous post-discharge survey. The parental survey had a sample size marginally adequate for statistical analysis, and the data did not allow matching by socioeconomic status, gender, or other potentially confounding factors. Another limitation is the study overlapped with the onset of COVID-19. To further assess the impact of COVID-19 on SFR implementation, additional research is needed to assess parent satisfaction in later SFR implementation (year 2 post-implementation) compared to early SFR implementation (year 1 post-implementation). Yet another limitation is that our study only captures the perspectives of parents and nurses. Future research can expand interviews to clinicians and administrators with diverse roles (e.g., advanced practitioners, physicians).

The SFR design offers numerous benefits including increases in privacy, respect for confidentiality, and eased facilitation of sterile and isolation procedures. Yet, the SFR design may have unintended consequences related to nurse visibility and nurse-parent communication compared to the OPBY design. Implementing SFR designs during COVID-19 or other biologic shocks may present additional challenges. Policies and procedures meant to keep families safe sometimes decrease parents’ perceived access to and responsiveness of the healthcare team. Supportive policies and procedures may need to accompany transitions to SFRs to realize improvements in parental assessment of quality of care. Supportive policies and procedures that may enhance parent confidence and trust involve increased nurse-parent communication, bedside rounds facilitating physician-parent communication, and additional parental supports such as more inclusive visitation policies.

## Supplementary information

Supplemental File

## Data Availability

The data are available from the authors by written request and approval of a data use agreement.

## References

[1] Martin JA, Hamilton BE, Osterman MJ, Driscoll AK. Births: final data for 2018. Natl Vital- Stat Rep. 2019;68:1–47. 32501202

[2] Walsh WF, McCullough KL, White RD. Room for improvement: nurses’ perceptions of providing care in a single room newborn intensive care setting. Adv Neonatal Care. 2006;6:261–70. 10.1016/j.adnc.2006.06.002. 17045946 10.1016/j.adnc.2006.06.002

[3] Stevens DC, Helseth CC, Thompson PA, Pottala JV, Khan MA, Munson DP. A comprehensive comparison of open-bay and single-family-room neonatal intensive care units at Sanford Children’s Hospital. HERD Health Environ Res Des J. 2012;5:23–39. 10.1177/193758671200500403 23224804

[4] Doede M, Trinkoff AM, Gurses AP. Neonatal intensive care unit layout and nurses’ work. HERD Health Environ Res Des J. 2018;11:101–18. 10.1177/1937586717713734 28627241

[5] Carter BS, Carter A, Bennett S. Families’ views upon experiencing change in the neonatal intensive care unit environment: from the ‘baby barn’ to the private room. J Perinatol. 2008;28:827–9. 18596717 10.1038/jp.2008.102

