# Supplementary material for: Nurse and parent perspectives of a neonatal intensive care unit redesign from open-bay to single-family rooms
Source: J Perinatol. 2025 Jul 3;45(6):851–6. doi: 10.1038/s41372-025-02342-w (PMC12263430; doi:10.1038/s41372-025-02342-w)
Supplement: Supplementary file 1 — Supplemental File [file 41372_2025_2342_MOESM1_ESM.docx]

**Supplemental File 1. Interview Protocol**

**Introduction**

1. Briefly tell us about yourself. What is your role on the NICU, and how long have you been in this role?

**Open-Bay Design**

1. Think back to when you worked on the open-bay NICU. Walk through what a typical shift was like.
2. What worked well when the NICU had an open-bay design?
   1. What didn’t work well?
3. When you worked in the open-bay NICU, how easy or difficult was it to…
   1. Communicate or collaborate with others?
   2. Get the support needed to do your job?
   3. Give patients the best quality care?
4. When you worked in the open-bay NICU, did you notice any differences in patient quality of care depending on patients’:
   1. Race or ethnicity
   2. Insurance status
5. In your opinion, what might explain these differences?
6. When you worked in the open-bay NICU, did you notice any differences in patient outcomes depending on patients’:
   1. Race or ethnicity
   2. Insurance status
   3. Whether their parents work full time
   4. Parent’s health literacy
7. In your opinion, what might explain these differences?
8. What were opportunities for improvement when the NICU had an open-bay design?

**Single-Family Design**

1. What is working well about the NICU’s single-family room design?
   1. What isn’t working well?
2. When you worked in the open-bay NICU, how easy or difficult was it to…
   1. Communicate or collaborate with others?
   2. Get the support needed to do your job?
   3. Give patients the best quality care?
3. Do you notice any differences in patient quality of care depending on patients’:
   1. Race or ethnicity
   2. Insurance status
4. In your opinion, what might explain these differences?
5. Do you notice any differences in patient outcomes depending on patients’:
   1. Race or ethnicity
   2. Insurance status
   3. Whether their parents work full time
   4. Parent’s health literacy
6. In your opinion, what might explain these differences?
7. If you were management, what changes – if any – would you make on the unit?
8. We recognize this is such a challenging time responding to COVID-19. In what ways has COVID-19 affected your work in general?

**Comparison Across Settings**

1. Single-family rooms are supposed to have positive outcomes, such as increased parent-neonate bonding time, and improved lighting and noise control. Do you believe these outcomes have equally benefited all patients on the NICU?
   1. Probe: If not, can you tell me more? Please explain.
   2. Probe: What patients benefited most from the transition to single-family rooms? How did they benefit?
   3. Probe: What patients benefited least from the transition to single-family rooms? What benefits weren’t realized?
2. What differences have you noticed between an open-bay vs. single-family room NICU?
   1. Probe: quality of care of patients on the NICU?
   2. Probe: health outcomes of patients on the NICU?
3. Are there processes that improved when the NICU transitioned to a single-family room?
   1. Probe: medication distribution procedure?
   2. Probe: milk bank?
   3. Probe: discharge process?
   4. Probe: quality improvement efforts?

**Wrap-Up**

1. Currently, how can management better support….
   1. your communication and collaboration with others?
   2. quality of care of patients on the NICU?
   3. health outcomes of patients on the NICU?
2. If you had unlimited resources to implement changes on the NICU, what would you want to do?
   1. What would you need to make this possible?
3. Is there anything else that you’d like to share with me?
4. What questions do you have for us?

**Supplemental File 2. Evidence / Direct Quotes from Nurse Interviews**

**Supplemental Table 1. Selected Direct Quote from Nurse Interviews Overall Quality of Care in Single-Family Room (SFR) Compared to Open-Bay (OPBY) Settings**

| **Context** | **Direct Quote** |
| --- | --- |
| A nurse provided detail related to the ability to get supplies and concluded the quality of care in the SFR was similar to that in OPBY. | “I mean, I feel like you’re still set up with everything. So having supplies close by, having everything in the room that you need. So I wouldn’t say [the NICU redesign] really impacted much to do with actual patient care. I mean, sometimes, yes, you might have to leave the room to get supplies if you’re out of bottles, or the NG tube feeding pieces. So sometimes you do have to, like, you know, stop what you’re doing for a minute to go get something. **But** **as far as actual patient care, I don’t really feel like it’s impacted much since we’ve moved,** which I’m grateful for. But I mean, I guess being physically distant from things makes it harder.” |

**Supplemental Table 2. Selected Direct Quotes from Nurse Interviews on Benefits of Single-Family Rooms (SFRs)**

| **Context** | **Direct Quote** |
| --- | --- |
| Benefits of increased privacy in SFRs included more space, opportunities for parent visitation, and facilitation of sterile and isolation procedures. | “The main thing is the privacy and the way you have as much room, as you have like each baby having their room, There’s so much room to do stuff. There’s so much privacy. The parents can stay overnight, they can stay whenever they want to. There’s some parents that stay literally 24/7 with their baby. Just in that room, just them and the baby. And if there’s a procedure or sterile procedure anything like that that needs to be done at the bedside, it can be done right there. You just shut the door. Everything’s kept sterile. So that works really well.” |
| Another benefit of  increased privacy in SFRs was the perceived comfort of parents to participate in breastfeeding and kangaroo, or skin-to-skin, care. | “Trying to get breastfeeding going is easier in the single-family rooms just because they feel like privacy and they are more comfortable. Just as far as furniture wise and privacy wise. I think it’s definitely helped families where the moms want to breastfeed the baby.” |
|  | “For the moms, even though there’s, you know, always curtains, but for the mom to do skin-to-skin and to pump, and to do all these things, it’s just, they have their own space and their own place to listen to just what they need to listen to, and not every other baby on the unit. So it just creates less stress, and it creates a more, optimal, space for the baby to mature all the ways that they couldn’t, you know, inside the womb.” |
| Nurses also reflected on how parents have commented on being more comfortable in the SFR setting. | “I’ve had parents tell me like they’ve had babies. Past NICU babies at the old hospital and then now I’ve had one at this hospital, and they expressed how much easier it is to come in and see your baby and have privacy, and you feel more comfortable in that setting, especially if they’re trying to breastfeed and do stuff like that, with their baby.” |

**Supplemental Table 3. Selected Direct Quotes from Nurse Interviews on Disadvantages of Single-Family Rooms (SFRs) and Strategies to Overcome Them**

| **Context** | **Direct Quote** |
| --- | --- |
| Increased privacy in SFRs resulted in a lack of nurse visibility, which presented communication challenges. | “With the private rooms, the parents cannot see that you’re busy doing something else, and sometimes they think that you’re not checking on them as often as you should when in reality we’re kind of caught up with another baby. And so that kind of makes it difficult, just because they can’t really see that you are busy. So they kind of just have to take your word on it and some parents understand, and then some parents don’t really understand.” |
|  | Another nurse described a parent shouting “my nurse hasn’t been in here, I don’t know . . . what the updates are on my baby.” |
|  | “Several families have been upset that their nurses weren’t promptly coming into the room when they get there.” |
|  | “And we hear that a lot now where we are now, where they come in and they go ‘My nurse wasn’t in here for over two hours.’ ” |
| Nurses discussed changing their communication in the SFRs to proactively mitigate parents’ negative perceptions and ensure that families understood that the quality of care remained high. | “But it’s easy if you let them know like: ‘Hey, I gotta go do this. I’ll be back in like 30 minutes, or whatever.’ You can try to give them a time frame like that, like kind of understand better. Or if you know you will be caught up, you can always call your buddy or somebody else to go in the room and let that family know like: ‘Hey your nurse will be back. She’s busy or whatever like that.’ I think… we can still provide the same quality of care, it’s just how well do you communicate that to the family.” |
|  | One nurse went into detail related to technological tools used to support communications as well as patient safety in the new SFRs. She said: “We try to educate them on the fact that our Voceras [voice-driven smartbadges allowing for hands-free communication among nursing staff] . . . are linked to the monitors, . . . and if an alarm goes off it . . . lets us know.” She also added: “If I have three children, I can pull up those other children’s monitors on each of the rooms that I’m in. So I may not be visible, but I can visually see all of my children’s actual cardiac monitors from any room I’m in.” |

**Supplemental Table 4. Selected Direct Quotes from Nurse Interviews on Impacts of the COVID-19 Pandemic**

| **Context** | **Direct Quote** |
| --- | --- |
| Implementation of bedside rounds changed during the COVID-19 pandemic. | “Part of this, I guess, has been COVID-related, but we used to do our rounds at the bedside at the old hospital. And unfortunately, it’s kind of hard to tell whether it was COVID, or the new building. But when COVID happened, right when we moved, we started doing the rounds in our conference room, so it was no longer in person. So that has been a different change, which I think has been hard for both bedside nurses and parents, because they can’t really see face to face, if that makes sense.” |
| Nurses shared  parental concerns about the access to other medical staff during the COVID-19 pandemic. | “Some parents get upset because they feel like they haven’t been updated by the provider since **we don’t do rounds in person**… I feel like some of the parents are upset because providers aren’t always at the bedside.” |
|  | “The providers are always available, but they’re not as visible, possibly as they used to be . . . [One mom] felt like she wasn’t getting updates [outside of nursing]. . . . I don’t know if they happen to go by at nine o’clock when they check the baby and the mom’s not in there. I don’t know what their process is about reaching out to that mom.” |
|  | Reflecting on the OPBY setting, a nurse stated: “Families were more visible like it was a smaller area . . . so if the provider walked up and down the hall, they could say, oh, my patient in that pod, I see them. Let me go touch base with them and give them an update.” |
| Policies restricted visitation in the early days of the COVID-19 pandemic. | “We first moved right before COVID. . . We were allowing you know more visitors to come in. They could be like the mom and the dad and then they could have like two other people, maybe. . . now it’s only the mom. It was the mom and the dad for a while and that was it. Now we have [extended] it to her mom and dad and one other person. The parents were upset about that and on [the] old unit before COVID, they could have their siblings come in.” |

**Supplemental Table 5. Selected Direct Quotes from Nurse Interviews on Disadvantages of the Open-Bay (OPBY) Setting**

| **Context** | **Direct Quote** |
| --- | --- |
| The OPBY setting had a lack of privacy for parents. | “If a patient was on isolation, that was a mess, because we had screens that we would put up, which you’re kind of like, ‘Is a screen gonna keep away whatever it is?’ You know, because you can’t talk about it with the other family. So they’re like, ‘What’s going on back there?’ Or if a patient died, obviously, that’s awful. And we tried to ask the families to step out. Or if it was going through a situation that it’s hard to have other families see what’s going on, or they see that the baby next to them might be dying. I mean, I don’t even know what we would have done if COVID happened over there. I have no idea what would have happened. So that would have been hard. I was thankful that that happened where we are now [in SFRs].” |
| In the OPBY setting, nurses implemented strategies to ensure that parents were not infringing on the privacy of other families. | “Sometimes we would have a problem with like other parents or other family members just kind of being nosy and just kind of like walking around the room looking at other babies and we had to remind them like you need to come in the pods and just go straight to your baby. Don’t be lingering around trying to look at other babies”. |

**Supplemental Table 6. Selected Direct Quotes from Nurse Interviews on Benefits of the Open-Bay (OPBY) Setting**

| **Context** | **Direct Quote** |
| --- | --- |
| One benefit of nurses being in the same room was increased parent confidence in the nursing care because parents could see the nurse providing care to other neonates when not providing care to their neonate. | “It was easy just because you were right there. …And you could clearly see if they were there and they needed help. You could just tell them, ‘Hey, I’ll be over there and …help you in a minute,’ especially if you were with another baby. And I feel like the parents felt like even if we were caring for another baby, they also felt like we were also watching their baby, but we were able to watch their baby, at the same time, so they felt kind of safe about leaving their baby, just because they knew that we were in the same room with their baby.” |
|  | “You pretty much knew everything that was going on, just because we’re all right there.” |
| Another benefit of nurses being in the same room was parental education. | “A good part too about that was if there was four babies in a room and I’m discharging a patient to a family, the family sitting in another space could hear what I’m saying and kind of absorb the discharge teaching that I’m telling them. And so they’ll get that on their discharge day, but they also get a little bit of information at that time, too.” |
